# Nest Success of Gunnison Sage-Grouse in Colorado, USA

**DOI:** 10.1371/journal.pone.0136310

**Published:** 2015-08-19

**Authors:** Amy J. Davis, Michael L. Phillips, Paul F. Doherty

**Affiliations:** 1 Department of Fish, Wildlife, and Conservation Biology, Colorado State University, Fort Collins, Colorado, United States of America; 2 Colorado Parks and Wildlife, Fort Collins, Colorado, United States of America; Cornell University, UNITED STATES

## Abstract

Gunnison Sage-Grouse (*Centrocercus minimus*) is a species of concern for which little demographic information exists. To help fill this information gap, we investigated factors affecting nest success in two populations of Gunnison Sage-Grouse. We assessed the relative effects of (1) vegetation characteristics (e.g., shrub height, shrub cover, grass cover, and grass height), (2) temporal factors (e.g., year, timing of incubation initiation, and nest age), (3) precipitation, and (4) age of the nesting female (yearling or adult) on nest success rates. We found 177 nests in the Gunnison Basin population (that contains 85–90% of the species) from 2005–2010 and 20 nests in the San Miguel population (that contains < 10% of the species) from 2007–2010. Temporal factors had the greatest impact on nest success compared to vegetation characteristics, precipitation, and female age. Nest success varied considerably among years ranging from 4.0%-60.2% in Gunnison Basin and from 12.9%- 51.9% in San Miguel. Nests that were initiated earlier in the breeding season had higher nest success (at least one egg hatches). Daily nest survival rates decreased during the course of incubation. None of the vegetation characteristics we examined were strongly related to nest success.

## Introduction

Nest success is one of the primary factors driving avian reproductive success, and thus population growth rates. Consequently, declines in nest success are thought to correspond to population declines for many bird species [1,2]. The range of Gunnison Sage-Grouse (*Centrocercus minimus*, GUSG) has contracted to 10% of the possible historic range [3,4] and GUSG are considered threatened under the U.S. Endangered Species Act [5]. Therefore, low nest success rates (defined as the probability of at least one egg hatching) for GUSG are of particular interest in terms of the status to the species and in aiding management of the species.

Understanding the relationship between habitat characteristics and environmental variation (e.g., vegetation structure, weather) and nest success can enable more informed and directed conservation of sage-grouse (e.g.,[6]). As ground-nesting birds, GUSG face a wide range of predators and generally rely on vegetation as concealment to protect their nests (e.g., [7,8]). Many ground-nesting bird species, including grouse, select nest sites that have greater concealment cover than the landscape in general (e.g., [9,10,11]). Previous research demonstrates that grouse select nest sites with lower densities of avian (visual) predators [12], but do not specifically select nest sites with lower risk from olfactory predators [13].

Research on the closely related Greater Sage-Grouse (*Centrocercus urophasianus*, GRSG) demonstrate variable relationships between vegetation cover and nest success. Several studies have found a positive relationship between shrub cover and nest success [14–18], while some have not found a relationship with shrub cover [19]. Other studies have found shrub height is more influential than shrub cover [18,20]. Grass cover [19,21,22] and grass height [14,21,23] were stronger indicators of nest success in a few studies. Forb cover has been examined in several studies but it has not been found to be strongly related to nest success [14,17,19,21,24].

Previous work investigating GUSG nest success has been limited in spatial or temporal extent and in sample size (<40 nests/year, [15,25,26,27]). The lack of information on GUSG nesting success coupled with the potential importance of nesting success to population viability spotlights the need for an in depth analysis of GUSG nesting success. We assessed the influence of percent shrub cover, average shrub height, percent grass cover, and average grass height with respect to nest success of GUSG. We predicted a positive relationship with each of these habitat characteristics and nest success because more vegetation will provide greater nest concealment. In addition to vegetation characteristics, we considered other factors that might correspond to changes in nest success rates. Some research shows that increased precipitation has a positive relationship with nest success [16,21], therefore we investigated the effect of annual precipitation on nest success. Previous research demonstrates that nest success of adult (after second year) females is greater than for yearling females [18], so we examined the effect of female age on nest success. Daily nest survival may be negatively correlated with nest age [17], therefore we also examined the potential effect of nest age. We also examined several temporal scales in which nest success might vary (e.g., yearly, within year). In summary, our objective was to assess the effect of vegetation characteristics, precipitation, female age, nest age, and time (i.e., year and week) on GUSG nest success.

## Methods

### Study Area

**Fig 1:**
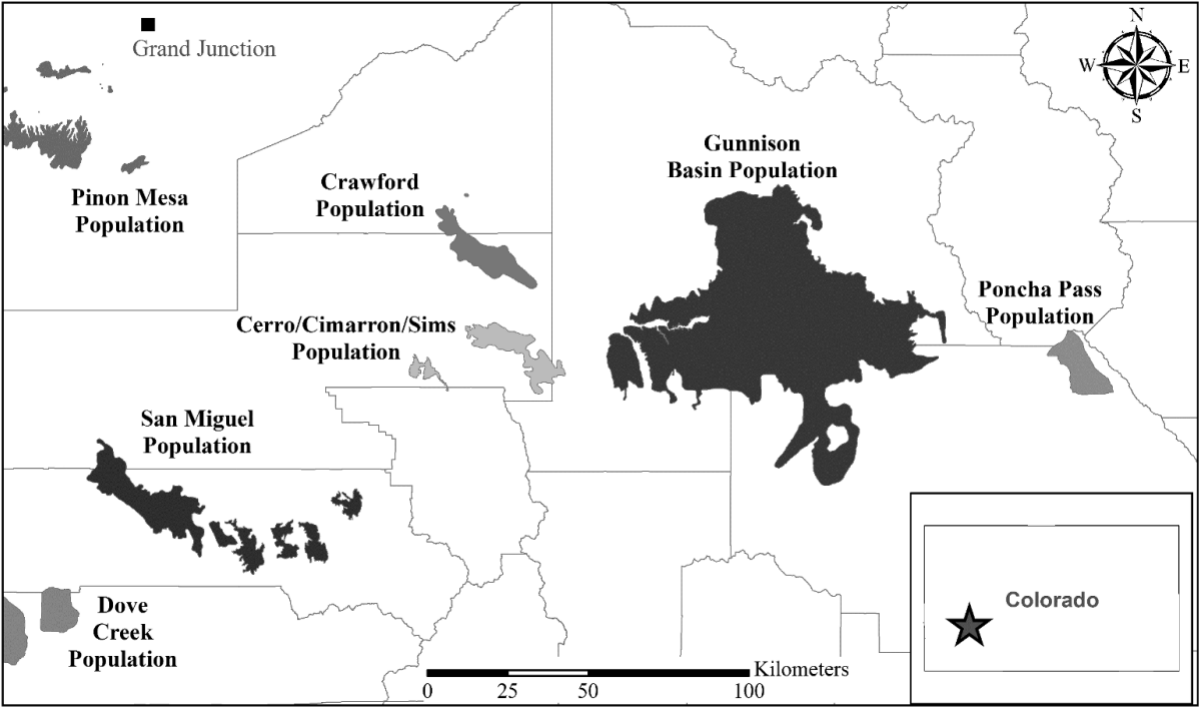
The distribution of seven isolated populations of Gunnison Sage-Grouse in Colorado, USA. The two study populations (Gunnison Basin and San Miguel) are shaded black. County boundaries are provided for reference.

Field methods were approved by the Colorado Parks and Wildlife Animal Care and Use Committee (#02–2005). The study species was not federally protected during the course of the study. Research was conducted with permission primarily on public land (U.S. Bureau of Land Management and U.S. Forest Service) and some private land with landowner permission. Our study area encompassed two of seven isolated populations of GUSG: Gunnison Basin and San Miguel. The Gunnison Basin population comprised 85–90% of all GUSG [4]. This population resided in the Gunnison Basin, in Gunnison and Saguache counties, Colorado, USA (Fig 1). Gunnison Basin is a 2,000 km^2^ intermontane basin ranging in elevation from 2,300 to 2,900m [28]. Mountainous terrain borders the north, east, and south-east sides of this population, and these areas contain habitat not commonly used by sage-grouse [6]. The western edge of the Gunnison Basin was not included in our study area due to access constraints. The San Miguel population is the second largest population (containing 3% of all GUSG, [4]) and therefore provided the highest chance of the six populations outside of Gunnison Basin of obtaining adequate sample sizes. The San Miguel population was located in Montrose and San Miguel counties, Colorado, USA. It was comprised of six, interconnected subpopulations over a 400 km^2^ area. Our study was conducted in the Miramont Reservoir subpopulation area of San Miguel. The elevation of this area ranged from 1,900 to 2,800m [4].

Both study areas were predominately sagebrush steppe dominated by sagebrush (*Artemisia* spp.). Gunnison Basin consisted of more mountain big sagebrush (*Artemisia tridentate*) interspersed with rabbitbrush (*Chrysothamnus* spp.), antelope bitterbrush (*Purshia tridentata*), serviceberry (*Amelanchier* spp.) and mountain snowberry (*Symphoricarpos oreophilus*). Black sagebrush (*A*. *nova*) was found on xeric ridge tops and south-facing slopes (Colorado Parks and Wildlife unpublished report, [28]). The vegetation characteristics varied among the six subpopulations within the San Miguel population. The shrubs in San Miguel were predominately low sagebrush (*Artemisia arbuscula*) and black sagebrush (*Artemisia nova*) with some mountain big sagebrush (*Artemisia tridentate*) along drainages (Colorado Parks and Wildlife unpublished report, [29]). Annual precipitation ranged from 20.3–34.2 cm during 2005–2010 in Gunnison Basin and ranged from 36.6–47.0 cm during 2007–2010 in San Miguel [30].

### Capture and Monitoring

We trapped GUSG using spot-lighting techniques [31,32] from 2005–2010 in the Gunnison Basin and 2007–2010 in San Miguel (mid-March through early-May). Captured grouse were fitted with a 17 g necklace-style radio transmitter (Advanced Telemetry Systems, Inc., Isanti, MN) and a numbered leg band (National Brand and Tag Company, Newport, KY). The transmitter weight was <2% of the average adult female GUSG’s body weight ($\bar{x}$ = 1270 g SD 90 g). We weighed each bird and used plumage characteristics to determine age [33,34]. Each GUSG was categorized as either an adult (second breeding season or later) or a yearling (first breeding season).

Females were tracked daily through the breeding season to ascertain nesting status. A female was determined to be nesting if she was located in the same place for ≥ 3 consecutive days. We recorded locations for the female at different times of day (0800–1700 hrs.). We used triangulation to estimate the location of the nest using maximum likelihood estimates in the triangulation software LOCATE II [35]. Visual observations of females on nests were avoided to minimize disturbing birds and avoid interfering with nesting behavior. Once a female was no longer at the nest, the nest was located to assess the fate (e.g., hatched, depredated, abandoned, or unknown) of the eggs. Nest scrapes were located for all presumed nesting sites. If a nest was abandoned or depredated, the female was tracked to ascertain if she re-nested.

### Vegetation Data

Vegetation characteristics were measured at all nest locations using techniques described by Canfield [36] and Daubenmire [37]. After the female moved from the nest, a 30 m transect was established, centered at the nest and oriented north-south. We estimated vegetation cover and height at 7 sample points along each transect (5 m intervals along the transect). A Daubenmire frame (20 x 50 cm) was used to visually estimate the percent cover of grasses to the nearest 5% at each interval [37]. We estimated shrub canopy cover using the line intercept method [36]. Shrub cover denotes cover including sagebrush and other shrub species (e.g., rabbitbrush and bitterbrush). We measured the height of the shrub closest to the Daubenmire frame at each sampling point. All vegetation data were collected by the same two individuals (M.L.P. and P.A.S) to minimize variability due to observer-based sampling error.

### Data Analysis

We used nest survival models [38] in Program MARK [39] to estimate rates of daily nest survival and examine the relationship between nest success and vegetation and temporal and individual covariates. We tested the covariates for correlations to ensure against multicollinearity. We evaluated the relative importance of each model using Akaike’s Information Criterion with a small sample size correction (AICc, [40]). We fit all additive combinations of factors to obtain our model set as suggested by Doherty et al. [41]. To evaluate the relative importance of each factor, we calculated the cumulative AICc weight (cumulative *w*_*i*_) associated with each variable [40]. This information theoretic approach allowed us to compare the strength of support for each covariate (larger values of cumulative *w*_i_ suggest strong support). For convenience, previous studies have adapted a cutoff of a cumulative *w*_i_ above 0.50 to be important [42] and we adopted this cutoff in our research.

The habitat factors we compared in our analysis included: percent shrub cover, average shrub height, percent grass cover, and average grass height. We compared the nest success rates between yearlings and adults. We examined the effect of annual precipitation for the 12 months preceding the nesting season on nest success. The longer a nest has been active the more visual and olfactory cues are expressed as the hen moves to and from the nest, which can increase the risk to the nest. Therefore, we modeled a linear relationship between daily nest survival and the length of time of incubation (hereafter ‘nest age’). We also examined several temporal covariates to address any unaccounted for variability in the data, including annual variability (‘year’), initiation timing (‘start week’), and an interaction between year and start week. The interaction between year and start week was intended to allow for breeding timing to vary with seasonal shifts from one year to the next.

Model averaged estimates of daily survival and parameter estimates were to be used if there was model uncertainty in the results [40]. The probability that a nest was successful was calculated as the product of the daily nest survival rates for the duration of incubation (typically 28 days). We used the delta method to estimate standard errors for nest success [43].

## Results

From 2005–2010 in Gunnison Basin and 2007–2010 in San Miguel, we tracked a total of 192 females (181 in Gunnison Basin and 11 in San Miguel). Thirty-nine of the females in Gunnison basin were yearlings and 138 were adults. All of the females in San Miguel were adults. We located 177 nests in the Gunnison Basin population and 20 nests in the San Miguel population with five and six renesting attempts respectively. All renesting attempts were by adult females. Average nest initiation varied by study population and year but not by female age. Across all years, average nest initiation date was 8 May (+-9.8 standard error) in Gunnison Basin and 20 May (+- 16.4 standard error) in San Miguel. In 2008 in Gunnison Basin, the average initiation was shifted to17 May (+- 8.4 standard error) corresponding with heavy snowfall that spring (n = 35). The latest average initiation in San Miguel was in 2010 at 1 June (n = 7).

Model uncertainty was considerable; thus, we employed model averaging for daily nest survival rates. Nest age, year, and start week were consistently in the top ranked models (Table 1) and had similarly large cumulative *w*_*i*_ (>0.99, Table 2). Cumulative *w*_*i*_ near one indicate strong support for a covariate, any covariates with cumulative *w*_*i*_ less than 0.5 are relatively not influential. The interaction between the year and start week was therefore considered important (cumulative *w*_i_ = 0.672).

**Table 1:** Gunnison Sage-Grouse nest success models from 2005–2010 in two populations in Colorado, USA. The top 10 models are shown as well as the null model for comparison (see Davis 2012 for the full model set). The Akaike’s Information Criterion (AIC_c_) for the top model was 925.622.

Model	Δ AIC_c_	*w*_*i*_ ^a^	K^b^
S(Year*Start week^c^ + Nest age)	0.000	0.133	19
S(Year + Start week + Nest age)	1.376	0.067	9
S(Year*Start week + Nest age + Shrub height)	1.770	0.055	20
S(Year*Start week + Nest age + Grass height)	1.958	0.050	20
S(Year*Start week + Nest age + % Grass cover)	1.984	0.049	20
S(Year*Start week + Nest age + Age)	1.989	0.049	20
S(Year*Start week + Nest age + % Shrub cover)	1.994	0.049	20
S(Year + Start week + Nest age + % Grass cover)	3.284	0.026	10
S(Year + Start week + Nest age + Age)	3.289	0.026	10
S(Year + Start week + Nest age + Shrub height)	3.337	0.025	10
S(.)	26.319	0.000	1

^a^ *w*_i_ are Akaike weights

^b^ K represents the number of parameters in each model

^c^ Start week = within-year incubation initiation timing

**Table 2:** Relative variable importance (cumulative Akaike Information Criterion (AIC_c_) weights—*w*_i_) for Gunnison Sage-Grouse nest success analysis for two populations in southwest Colorado, USA from 2005–2010.

Covariate	Cumulative variable *w*_i_
Start week^a^	1.000
Nest age	0.999
Year	0.999
Year*Start week	0.670
Shrub height	0.291
% Grass cover	0.274
% Shrub cover	0.273
Grass height	0.273
Age	0.273
Precipitation	0.000

^a^ Start week = within-year incubation initiation timing

**Fig 2:**
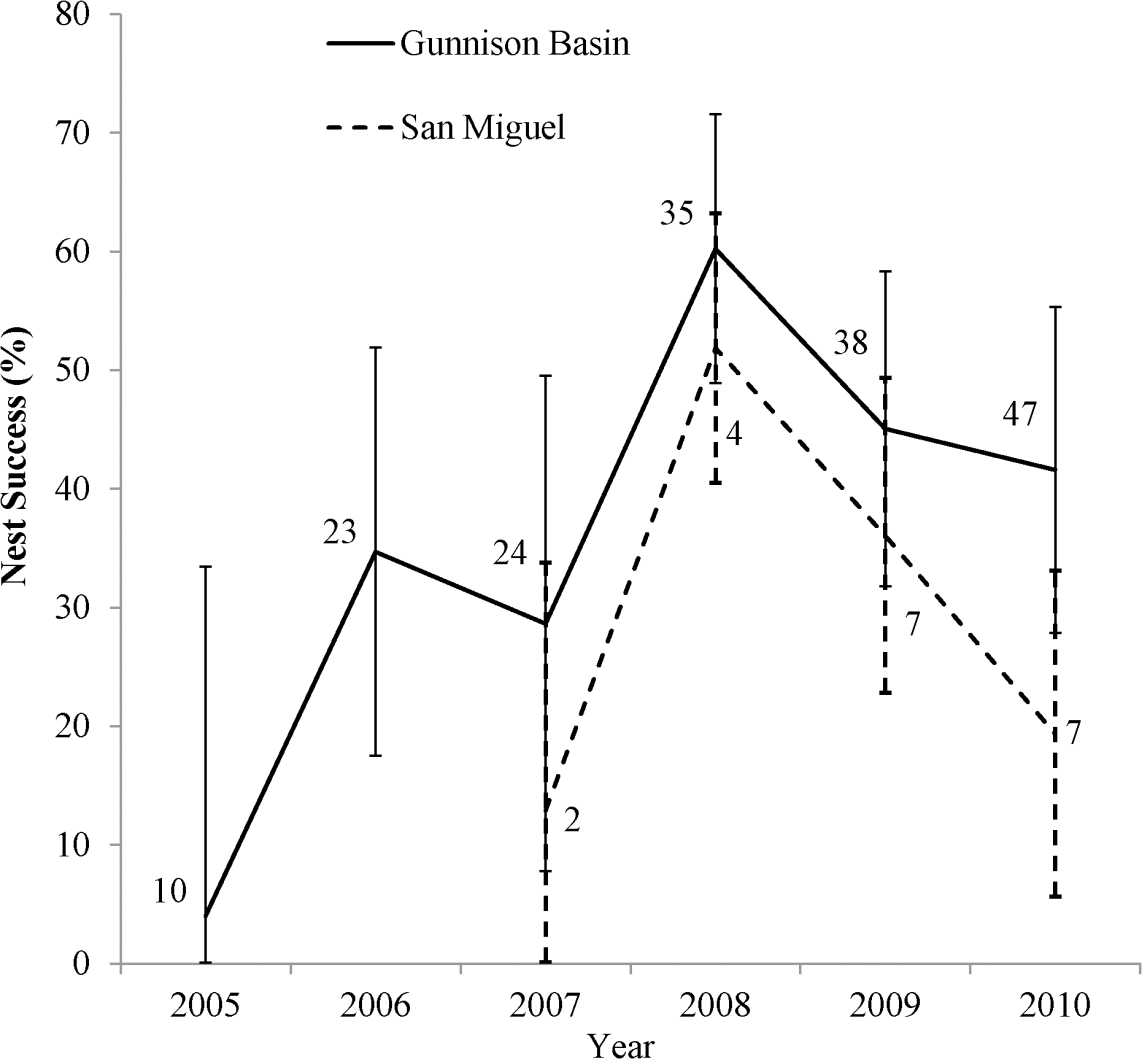
Nest success rates (%) for Gunnison Sage-Grouse. Nest success estimates by year (2005–2010) and population (Gunnison Basin and San Miguel) for Gunnison Sage-Grouse in Colorado, USA, with 95% confidence intervals. The estimates are based on model averaging and sample sizes are by year.

**Fig 3:**
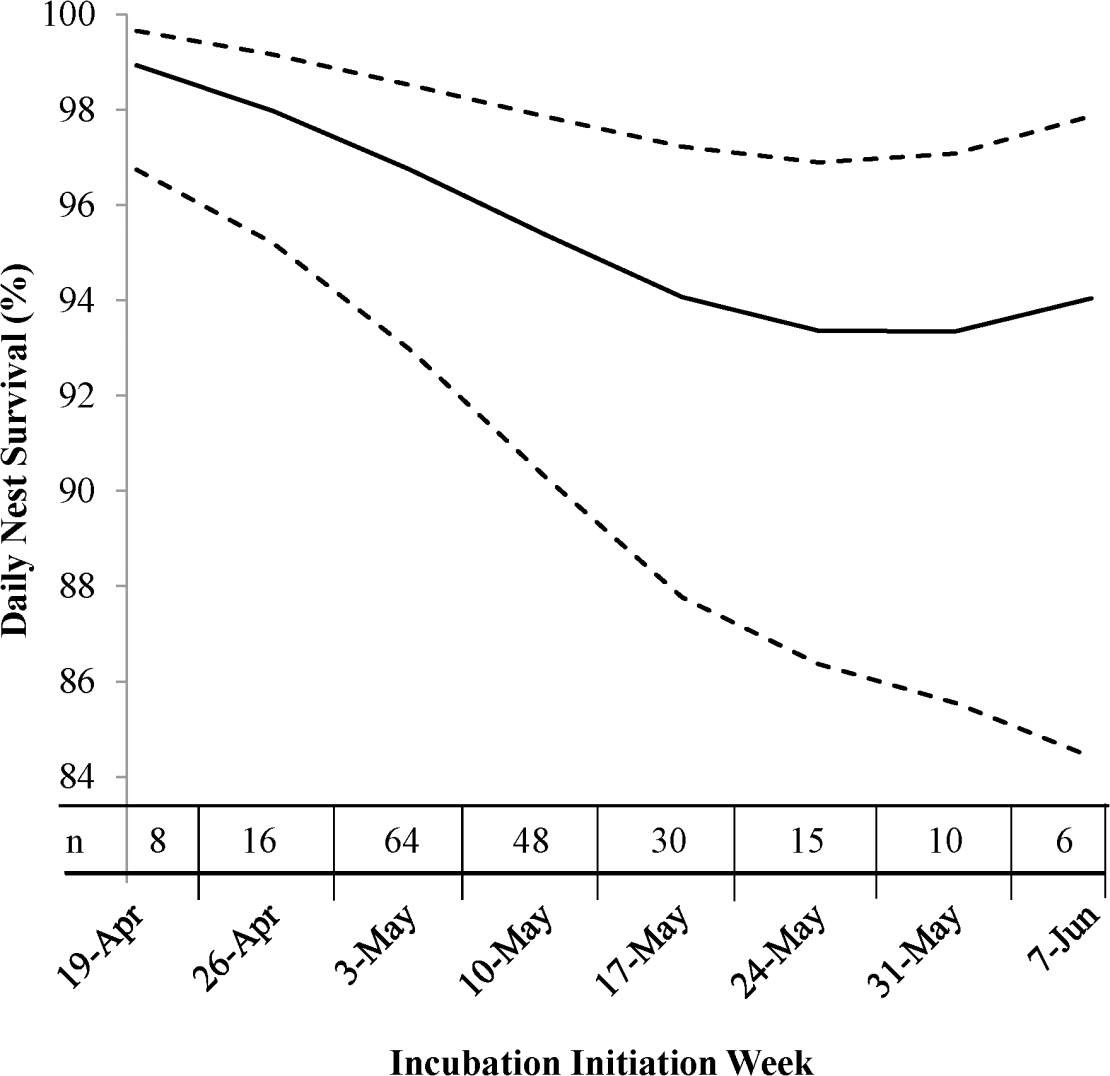
Daily nest survival rates (%) for Gunnison Sage-Grouse by incubation initiation (start week) for two populations in southwest Colorado, USA from 2005–2010. Estimates are from the model that includes year, start week, and nest age. 95% confidence intervals are shown as dashed lines and sample size by start week are shown on the x-axis.

Due to the small sample size in San Miguel comparing the Gunnison Basin and San Miguel populations was compromised. However, there were differences in nest initiation timing (start week) between the two populations and differences in within-year timing of nesting was a strong factor in our analysis. Therefore, our nest success rates are estimated by year and population calculated based on the corresponding average start week (Fig 2). In Gunnison Basin, nest success fluctuated among years from a low in 2005 of 4.0% to a high in 2008 of 60.2% (Fig 2). Nest success estimates varied in San Miguel from a low in 2007 of 12.9% to a high in 2008 of 51.9%. Within each year, nests initiated early in the breeding season had greater success than those initiated later (Fig 3). Daily survival rate of a nest decreased as incubation progressed (*β*_*nest age*_ = -0.05, 95% CI: -0.11, -0.04).

**Fig 4:**
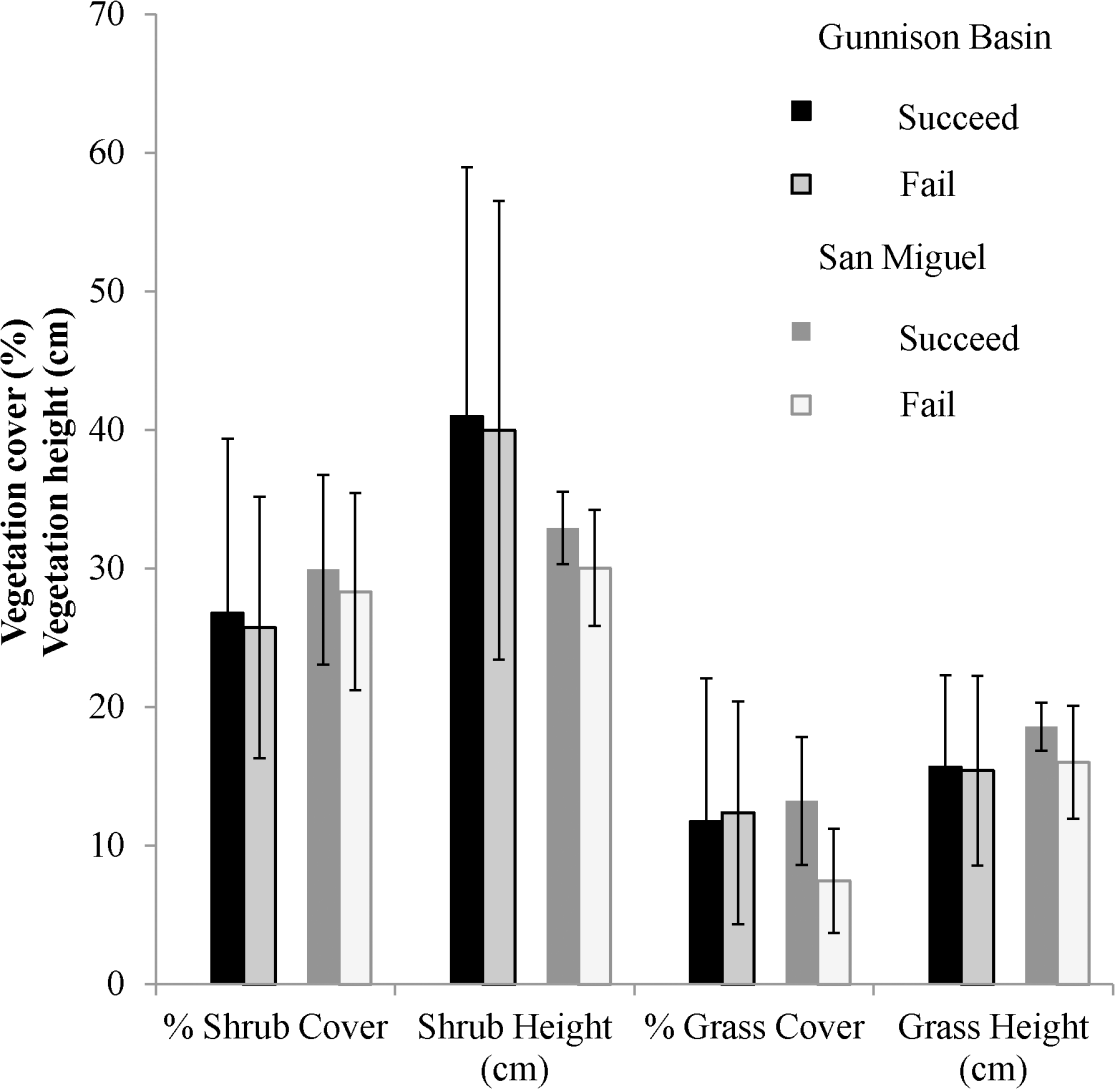
Average vegetation measurements (with standard errors) for nests in the Gunnison Basin population (n = 177) and the San Miguel population (n = 20) of Gunnison Sage-Grouse in Colorado, USA. Data are separated by nests that succeeded (i.e., eggs hatched) and nests that failed from 2005–2010.

Vegetation characteristics at nests were similar between the populations (Fig 4). We did not find any of the vegetation covariates to be closely related to nest success (cumulative *w*_i_ <0.5; Table 2). Additionally, the confidence intervals of the vegetation covariates overlapped zero (*β*_*% shrub cover*_ = 0.089, 95% CI: -0.749, 1.002; βshrub _*height*_ = -0.002, 95% CI: -0.010, 0.005; *β*_*% grass cover*_ = -0.001, 95% CI: -0.010, 0.008; *β*_*grass height*_ = 0.002, 95% CI: -0.012, 0.016) suggesting a limited effect.

Annual precipitation had no relative support (cumulative *w*_i_ < 0.001, Table 2). Age of the nesting female was not an important factor (cumulative *w*_i_ = 0.273, Table 2). Nest initiation rates were slightly higher for adults (0.93, SE = 0.02) than yearlings (0.89, SE = 0.04) in Gunnison Basin. Adult nest initiation rates were slightly lower in San Miguel than Gunnison Basin (0.88, SE = 0.08); however, no yearlings were captured in San Miguel and thus we could not make a comparison with yearlings in the Gunnison Basin.

## Discussion

Gunnison Sage-Grouse nest success varied considerably from year to year (4% to 60%, with an average of 35%). Temporal covariates dominated the results with the year in which a bird nested and the timing of the nesting within that year influencing the probability of nest success. These temporal covariates may have accounted for factors that we did not explicitly examine, such as combinations of weather conditions, other habitat metrics, or predator densities and/or predator demands on grouse (e.g., variations in availability of alternate prey). For instance, the 2007–2008 winter in Colorado was extremely harsh with heavy snowfall that persisted late into spring [30]. However, the spring of 2008 exhibited the highest nest success rate during our study. There are several potential explanations for our finding: 1) the higher nest success rate might have been the result of better vegetation quality due to the increased spring moisture [44], 2) the success could be due to less predation pressure due to the availability of other prey sources such as winter killed ungulates (Colorado Parks and Wildlife unpublished data, [45,46,47]), 3) the harsh winter may have led to fewer predators or reduced predator fitness [48]. The importance of temporal effects highlights the uncertainty in the biological factors that contribute to nest success for GUSG. Therefore, monitoring some of these other factors (e.g., predator densities, habitat quality) could be beneficial to more fully understanding nest success of GUSG.

Precipitation is related to habitat quality for sagebrush habitats [44]. Increased annual precipitation has been related to increases in nest success for GRSG on a daily scale [16], a one-day lag at the daily scale [19] and on an annual scale [21]. The localized nature of precipitation in the mountains of Colorado makes it unlikely that the daily precipitation measurements would reflect precipitation at individual nest sites across the study areas. Therefore, we examine an annual impact of precipitation on nests and not a daily impact. Annual precipitation was not related to nest success in our study. This may be due, in part, to the fact that San Miguel received ~40% more rain annually than Gunnison Basin [30] and a direct correlation between precipitation and nest success without accounting for population is weak. The temporal variables may be accounting for precipitation and other weather effects that contribute to changes in nest success.

The age of the nest was an important determinant of daily survival rates for GUSG in our study. Previous work has found the relationship between daily nest survival and nest age to be positive (Mountain Plover, *Charadrius montanus*) [38] or negative (GRSG, [17]) depending on the species. A positive effect might indicate high abundance of generalist predators that quickly depredate the most vulnerable nests [38]. Similar to our study, Kolada et al. [17] found a negative relationship between nest age and daily survival rates for GRSG in California. Coates and Delehanty [49] monitored incubation patterns of GRSG and found that incubation constancy (percentage of time spent at the nest in a 24-hour period) was lower as incubation progressed. An increase in movement to and from a nest might increase the chance a predator will be alerted to the nest’s location. This could contribute to the negative relationship we found between daily nest survival and nest age.

We focused on four vegetation characteristics which have been found to be important indicators of nest success in previous studies: percent shrub cover [14,16,17], average shrub height [18,20], percent grass cover [19,21,50], and grass height [14,16,21,23,50] around the nest. However, we found none of these vegetation covariates to be strongly correlated with nest success. Specific guidelines for habitat management for GUSG detail optimal ranges of vegetation cover for nesting [4] using data from Young [15] and Apa [25]. The ranges of shrub cover and shrub heights that we observed were similar to those outlined in the management guidelines. Although we did not detect an effect of vegetation characteristics on nest success, we caution against ignoring the important role these features may have in GUSG life history. Vegetation characteristics are important in nest site selection (e.g., [9,10,11]). The fact our nests had measurements within the guidelines may have made discerning vegetation effects more difficult than if they varied outside those ranges.

The variability of factors impacting nest success in previous work on GRSG suggests forces may be acting on each population differently. Johnson et al. [51] determined that optimal management strategies for a species were different among populations when variability existed between the vital rates of those populations. The two populations we examined differ in their population size [4], and inhabit areas with differing vegetation characteristics and weather patterns [30]. Therefore, a difference might be expected in the dynamics of the two populations (e.g., the effects of demographic and environmental stochasticity). Unfortunately the samples size in the San Miguel population prevented us from comparing the populations. Estimates were calculated by population and year to reflect the differences in the respective timing of nest initiation (Fig 2). Peak nesting was later in San Miguel and within year trends showed lower nest success corresponded later nest initiation (Fig 3). However, a larger sample size in San Miguel would allow a direct comparison between the populations which would be beneficial for implementing effective management in the two populations.

Generally, female age can influence nest initiation rates, nest success rates, renesting rates, and ability to rear chicks. Adults and yearlings in our study had similar nest success rates, but adults tended to initiate nests more than yearlings, as in Connelly et al. [52]. Interestingly, no yearlings were observed in the San Miguel populations, suggesting that recruitment was low in this population [53].

Previous to this study, information on the nest success rates of GUSG, how nest success rates varied over time, and which factors impact those rates was unavailable. Our study shows that nest success of GUSG can vary considerably over time. Therefore caution should be used when evaluating impacts to GUSG nest success at small temporal scales. Vegetation characteristics are often targets for management actions to help conserve GUSG. Although vegetation characteristics were not strong indicators of nest success in our study, the majority of nests had vegetation characteristics that were within the guidelines for suitable nesting habitat for GUSG [4]. The impact of vegetation may be stronger in nest selection than in nest success for this species.

## Supporting Information

S1 Dataset Nest success data for Gunnison Sage-Grouse. Table of nesting information for Gunnison Sage-Grouse in Gunnison Basin (2005–2010) and San Miguel (2007–2010) populations.
(XLSX)
